# Trends in Self-reported Forgone Medical Care Among Medicare Beneficiaries During the COVID-19 Pandemic

**DOI:** 10.1001/jamahealthforum.2021.4299

**Published:** 2021-12-30

**Authors:** Sungchul Park, Jim P. Stimpson

**Affiliations:** 1 Department of Health Management and Policy, Dornsife School of Public Health, Drexel University, Philadelphia, Pennsylvania

## Abstract

**Importance:**

Evidence suggests that the COVID-19 pandemic has had a negative association with medical care access. As prior studies mainly focused on the initial stage of the COVID-19 pandemic, less is known about how trends in forgone medical care changed over time.

**Objective:**

To examine trends in and reasons for forgone medical care among Medicare beneficiaries during the COVID-19 pandemic.

**Design, Setting, and Participants:**

This cross-sectional study analyzed Medicare beneficiaries using data from 3 waves of survey data from the Medicare Current Beneficiary Survey COVID-19 Supplement (Summer 2020, Fall 2020, and Winter 2021). Data analyses were conducted between July and August 2021.

**Exposures:**

Date of interview.

**Main Outcomes and Measures:**

Self-reported forgone medical care because of COVID-19.

**Results:**

A total of 23 058 Medicare beneficiaries were included (13 005 women [56.4%]; 10 445 [45.3%] 75 years old and older). The survey response rates for each wave were 72.6%, 78.95, and 79.6%, respectively. The rates of reported forgone medical care because of COVID-19 decreased from the week of June 7, 2020, to the week of April 4 to 25, 2021, but the largest difference in the rates was found between June 7 and July 12, 2020 (22.4% to 15.9%). Physician-driven factors accounted for about 70% of the forgone medical care. The proportion of those who reported forgoing medical care because of physician-driven factors tended to decrease from 66.2% in the week of July 7, 2020, to 44.7% in the weeks of April 4 to 25, 2021. The likelihood of forgone medical care was 4 percentage points (95% CI, 0.03-0.05) higher among those who reported feeling more stressed or anxious than those who did not, 3 percentage points (95% CI, 0.01-0.04) higher among those who reported feeling more lonely or sad than those who did not, and 3 percentage points (95% CI, 0.01-0.04) higher among those who reported feeling less socially connected than those who did not.

**Conclusions and Relevance:**

The results of this cross-sectional survey study suggest that the COVID-19 pandemic may exacerbate existing barriers to care and lead Medicare beneficiaries to delay needed care. Policy makers must continue to identify effective means of meeting the forgone care backlog and maintaining continuity of care, especially for those with mental health problems.

## Key Points

**Question:**

How did forgone medical care among Medicare beneficiaries change during the COVID-19 pandemic?

**Findings:**

In this cross-sectional survey study of 23 058 Medicare beneficiaries using 3 waves of data collection during the COVID-19 pandemic (Summer 2020, Fall 2020, and Winter 2021), patient-reported forgone medical care decreased over time, but the largest decrease was observed in Summer 2020. Most of the forgone medical care was associated with physician-driven factors, and forgone medical care was more common among those who reported mental health problems.

**Meaning:**

The study results suggest that Medicare beneficiaries experienced limited care access during the initial stage of the COVID-19 pandemic but have improved care access over time.

## Teaser

This survey study examines trends in and reasons for forgone medical care because of COVID-19 among Medicare beneficiaries during the COVID-19 pandemic.

## Introduction

Before 2020, delayed or forgone medical care was an ongoing health policy issue that was associated with poor health outcomes that inequitably affected vulnerable populations.^1,2,3,4,5^ There were reports of disparities in forgone care associated with sex, race and ethnicity, rural residence, and financial hardship status.^1,5,6^ Also, people with chronic health conditions are more likely to forgo needed treatments, especially if the physician’s office presents logistical barriers, such as lack of care coordination and inconvenient clinic hours.^4,6^ Preliminary reports suggest that the COVID-19 pandemic adversely affected medical care access.^7,8^ About 40% of US adults reported forgone medical care during the COVID-19 pandemic, citing fear of COVID exposure among the reasons.^9^ Therefore, COVID-19 may have exacerbated prepandemic predictors of forgone care.^3,4^

In association with this forgone care, preliminary reports suggest that there is an increased risk of spillover effects for negative patient outcomes that is associated with the COVID-19 pandemic.^10,11,12,13,14^Among the spillover effects of the pandemic are changes in mental health.^15^ The share of US adults who reported depressive symptoms, substance use, suicidal ideation, and psychiatric disorder substantially increased during the pandemic.^16,17,18,19,20^ The social isolation and lack of access to medical or behavioral health care from the pandemic may be among the factors associated with negative mental health outcomes.^15^ Mental health has been identified as a predictor of unmet or forgone medical care before the pandemic.^21^ Therefore, it is important to consider how the association of the COVID-19 pandemic with mental health problems may contribute to forgone medical care.^1,2,15^

Most studies of what is currently known are based on surveys during the initial stage of the COVID-19 pandemic, with limited information from respondents since the widespread administration of vaccines. These early studies about forgone care were likely associated with supply side factors, as offices were shut and hospital beds were at capacity with patients with COVID-19 and health systems lacked medical supplies and staff. There is limited information about the factors that might have contributed to forgone care after health systems regained the capacity to treat patients. Moreover, there is limited understanding of the experiences of Medicare beneficiaries who have high needs for medical care and can elucidate factors that contribute to forgone care other than lack of health insurance.

In this study, we examined trends in patient-reported forgone medical care because of COVID-19 among Medicare beneficiaries in 3 waves of data collection during the COVID-19 pandemic (Summer 2020, Fall 2020, and Winter 2021). Specifically, we conducted 4 analyses of forgone care informed by the Andersen model of health care utilization.^21^ First, we expected that forgone medical care because of COVID-19 decreased over time overall and by types of services commensurate with decreased cases of the virus. Second, we expected that reasons for forgone medical care associated with physician factors (eg, lack of physician appointments) and patient factors (eg, transportation) that were demonstrated in the research literature before the pandemic would be exacerbated because of COVID-19.^5,7,8,9^ Third, we expected that forgone medical care was associated with mental health problems during the COVID-19 pandemic, consistent with the association that has been demonstrated in the research literature before the pandemic for persons with chronic conditions.^4,6^ Finally, we expected that forgone medical care was more prevalent among those who reported mental health problems over time.

## Methods

### Data

We used 3 waves of survey data from the Medicare Current Beneficiary Survey (MCBS) COVID-19 Supplement Public Use File, collected via a telephone survey in Summer 2020 (June 7 to July 12), Fall 2020 (October 4 to November 8), and Winter 2021 (February 28 to April 25). The MCBS is a nationally representative survey of all Medicare beneficiaries. The original MCBS combined information from Medicare claims and administrative data with an interview survey. To better understand how the COVID-19 pandemic affected Medicare beneficiaries, the MCBS COVID-19 Supplement was administered to existing MCBS sampled beneficiaries who were living in the community as a test of the COVID-19 rapid response protocol. The survey was conducted in either English or Spanish. The MCBS COVID-19 Supplement has been used in prior studies.^22,23^

This study was exempted from review and informed consent by the institutional review board of Drexel University as the data were deidentified and publicly available. Reporting followed the Strengthening the Reporting of Observational Studies in Epidemiology (STROBE) reporting guideline.

### Study Sample and Variables

The study sample included community-dwelling Medicare beneficiaries who were interviewed in Summer 2020, Fall 2020, or Winter 2021 and had complete information. The outcome variable was (self-reported) forgone medical care because of COVID-19. We created a binary indicator for forgone medical care because of COVID-19. The primary outcome variable was any medical care reported to be forgone because of COVID-19, but we also included 8 service-specific outcomes (urgent care, surgery, diagnostics, prevention, checkup, dental, vision, and hearing). Also, we included reasons for forgone medical care as a secondary outcome variable. We created a categorical variable for reasons reported for forgoing planned care because of COVID-19. We included the following 8 reasons: 4 patient-driven factors (patient had no transportation, patient wanted to stay at home, patient felt risk, or patient had other reasons,) and 4 physician-driven factors (physician’s office was closed, physician had other priorities, physician reduced appointments, or physician had other reasons). Participants were allowed to report multiple reasons for forgone medical care because of COVID-19.

The key independent variables were the date of interview and mental health status. First, we created a categorical variable indicating 18 interview dates (6 interview dates for each survey wave). The unit of time was a week. Second, we created a binary indicator for mental health status. We included 3 measures of mental health status during the COVID-19 pandemic compared with the prepandemic period (stress/anxiety, loneliness/sadness, and social connection) (eAppendix in the Supplement). We categorized responses as more or not (including about the same or less) for stress/anxiety and loneliness/sadness and as less or not (including about the same or more) for social connection. These measures were adapted from the Research and Development Survey at the National Center for Health Statistics.^24^

To adjust for differences in sample characteristics, we selected predisposing, enabling, and need factors based on the Andersen model of health care utilization.^25^ Predisposing factors included age, sex, and self-reported race and ethnicity. Enabling factors included income, urban residence, census region of residence, dual eligibility for Medicare and Medicaid, use of a language at home other than English, ability to access basic needs during the pandemic (paying rent/mortgage, getting medication, getting food wanted, or getting home supplies), and access to telehealth. Need factors included self-reported 13 health conditions and smoking status.

### Statistical Analysis

We first estimated descriptive statistics for the sample. We then estimated adjusted outcomes using regression models. To examine trends in forgone medical care among Medicare beneficiaries during the COVID-19 pandemic, we first conducted a linear probability model on forgone medical care while controlling for demographic and socioeconomic characteristics, health status variables, and the date of interview. Then, we estimated the adjusted rates of the outcome by date of interview. This allowed for comparison of the outcome variable across groups that were similar across the set of control variables. There may have been differences in coverage by dual eligibility status, possibly leading to differences in access to care. Thus, we limited the sample to non–dual Medicare beneficiaries and performed the analysis previously described.

To understand the underlying mechanism for forgone medical care during the pandemic, we examined the reported reasons for forgone medical care because of COVID-19 by date of interview. We reported as a (unadjusted) proportion for each of the 8 factors. To examine whether forgone medical care was partly attributable to mental health problems among Medicare beneficiaries during the COVID-19 pandemic, we conducted a linear probability model on forgone medical care while controlling for variables described previously as well as 3 measures of mental health status. To examine whether forgone medical care was more pronounced among those with mental health problems over time, we conducted a linear probability model on forgone medical care while controlling for the variables described previously, as well as each measure of mental health status and the interaction term between each measure of mental health status and the date of interview. We then estimated the adjusted rates of the outcome by date of interview and mental health status. For all analyses, we used a complex sample design with sampling weights provided by the MCBS to produce nationally representative estimates. Analyses were conducted using Stata, version 16.1 (Stata Corp), and the statistical significance level was set at .05.

## Results

The final sample included 23 058 Medicare beneficiaries (13 005 women [56.4%]; 10 445 [45.3%] 75 years and older) (Table 1). The survey response rates for each wave were 72.6%, 78.95, and 79.6%, respectively. We found that 11.5% of Medicare beneficiaries reported forgone medical care because of COVID-19. Dental care was the most common type of care that Medicare beneficiaries delayed or did not receive because of the pandemic (4.3%), followed by prevention (4.0%) and checkup (3.9%). A substantial portion of Medicare beneficiaries experienced mental health problems during the pandemic. Nearly 40% of Medicare beneficiaries reported feeling more stressed or anxious, 21.5% reported feeling more lonely or sad, and 37.2% of Medicare beneficiaries reported feeling less socially connected during the pandemic. Full sample characteristics are presented in the eTable in the Supplement.

**Table 1.** Descriptive Statistics of Key Variables Among the Surveyed Medicare Beneficiaries

Variables	Beneficiaries, No. (%)
No.	23 115
Forgone medical care	
Any care	2661 (11.5)
Urgent care	140 (0.6)
Surgery	430 (1.9)
Diagnostics	769 (3.3)
Prevention	932 (4.0)
Checkup	912 (3.9)
Dental	992 (4.3)
Vision	605 (2.6)
Hearing	144 (0.6)
Mental health status	
More stressed or anxious	9178 (39.7)
More lonely or sad	4964 (21.5)
Less socially connected	8605 (37.2)
**Interview date**	
Week of	
June 7, 2020	1859 (8.0)
June 14, 2020	2684 (11.6)
June 21, 2020	1539 (6.7)
June 28, 2020	973 (4.2)
July 5, 2020	682 (3.0)
July 12, 2020	297 (1.3)
October 4, 2020	1293 (5.6)
October 11, 2020	1746 (7.6)
October 18, 2020	1285 (5.6)
October 25, 2020	1154 (5.0)
November 1, 2020	924 (4.0)
November 8, 2020	486 (2.1)
February 28, 2021	2493 (10.8)
March 7, 2021	2539 (11.0)
March 14, 2021	1378 (6.0)
March 21, 2021	711 (3.1)
March 28, 2021	453 (2.0)
April 4-25, 2021	619 (2.7)

**Figure 1.**
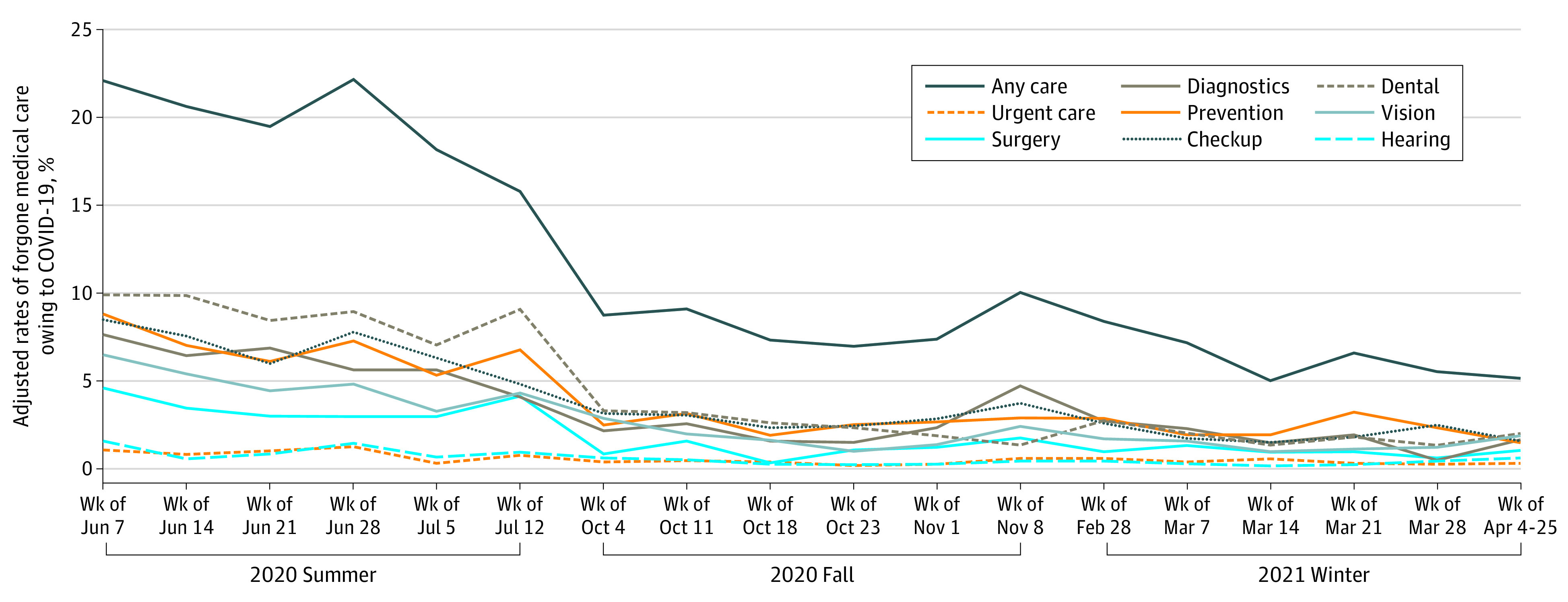
Adjusted Rates of Forgone Medical Care Because of COVID-19 Reported Among Medicare Beneficiaries by Date of Interview Adjusted rates were estimated using a linear probability model on forgone medical care while controlling for age, sex, race and ethnicity, income, rural residence, census region of residence, Medicaid eligibility, use of a language at home other than English, access to telemedicine, ability to access basic needs during the COVID-19 pandemic, self-reported health conditions, and the date of interview. Then, we estimated the adjusted rates of the outcome by date of interview. For all analyses, we accounted for complex sample design with sampling weights provided by the Medicare Current Beneficiary Survey (MCBS) to generate nationally representative estimates.

The adjusted analysis showed the rates of forgone medical care because of COVID-19 decreased from the week of June 7, 2020, to the week of April 4 to 25, 2021, but the largest difference in the rates was observed between June 7 and July 12, 2020 (22.4% [95% CI, 20.0%-24.6%] during the week of July 7 to 15.9% [95% CI, 11.1%-20.5%] during the week of July 12) (Figure 1). The rates of forgone medical care also decreased from October 4, 2020, to April 25, 2021, but the magnitude of the difference was relatively modest (8.8% [95% CI, 7.0%-10.6%] to 5.2% [95% CI, 3.3%-7.2%]). Overall, a similar phenomenon was found in all types of service-specific care. We found that unadjusted rates of reporting forgone medical care because of COVID-19 were similar to the adjusted rates (eFigure 1 in the Supplement). Also, the results remain similar when we limited the sample to non–dual Medicare beneficiaries (eFigure 2 in the Supplement).

**Figure 2.**
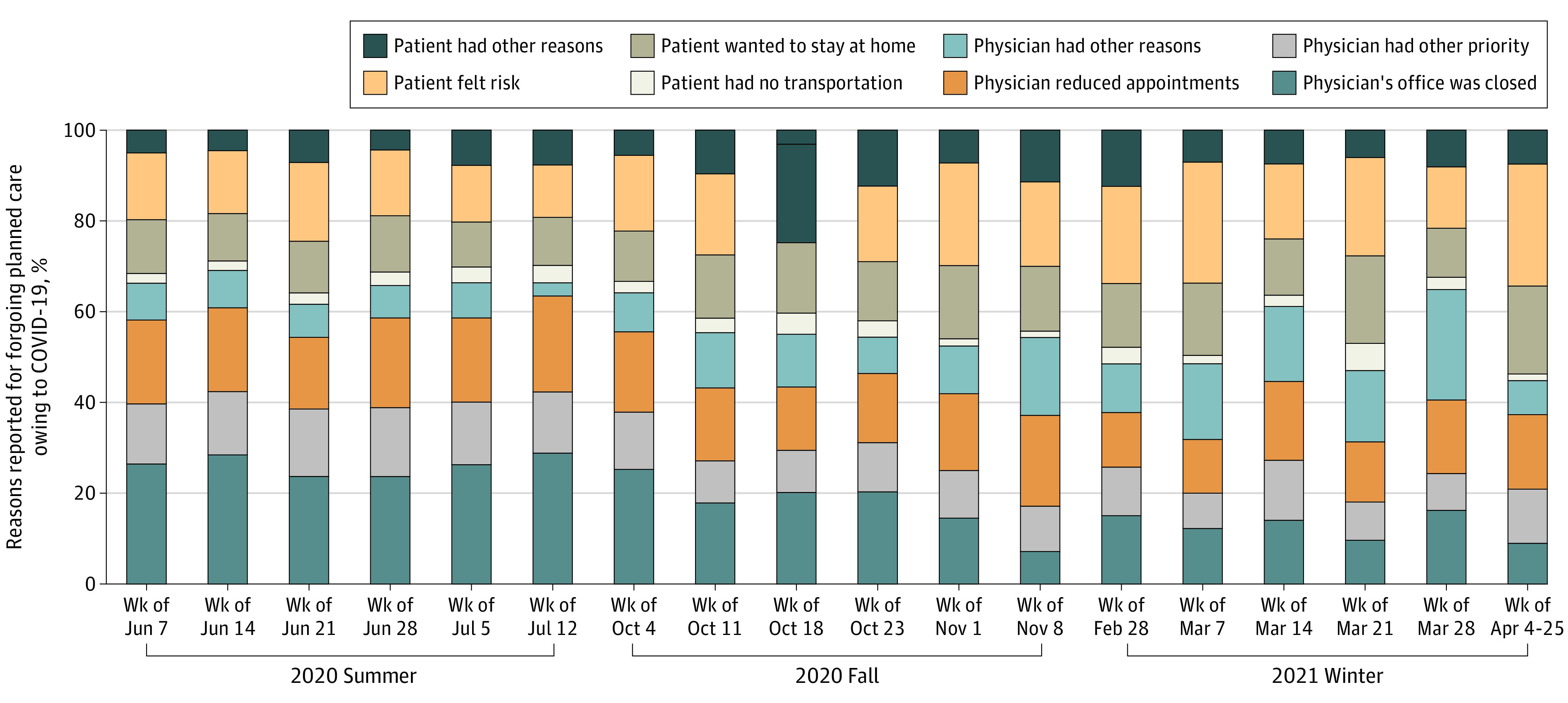
Reasons Reported for Forgoing Planned Care Because of COVID-19 Among Medicare Beneficiaries by Date of Interview Participants were allowed to report multiple reasons for forgone medical care because of COVID-19.

The unadjusted analysis showed that most of the forgone medical care was associated with physician-driven factors, which accounted for about 70% of forgone medical care (Figure 2). However, the proportion of those who forwent medical care because of physician-driven factors tended to decrease from 66.2% during the week of July 7, 2020, to 44.7% during the weeks of April 4 to 25, 2021. In Summer 2020, the most common physician barrier was that the physician’s office was closed. By April 4 to 25, 2021, the most common physician barrier was that the physician had reduced appointments. The most common patient-reported factors were that the patient felt risk and wanted to stay home.

The logistic regression suggested that forgone medical care was partly explained by mental health problems (Table 2). The likelihood of forgone medical care because of COVID-19 was 4 percentage points (95% CI, 0.03-0.05) higher among those who reported feeling more stressed or anxious than those who did not, 3 percentage points (95% CI, 0.01-0.04) higher among those who reported feeling more lonely or sad than those who did not, and 3 percentage points (95% CI, 0.01-0.04) higher among those who reported feeling less socially connected than those who did not.

**Table 2.** Results From a Linear Probability Model of Forgone Medical Care Reported by Medicare Beneficiaries

Variables	Forgone medical care because of COVID-19	
	Percentage point (95% CI)	*P* value
Mental health status		
More stressed or anxious	0.04 (0.03 to 0.05)	<.001
More lonely or sad	0.03 (0.01 to 0.04)	.001
Less socially connected	0.03 (0.01 to 0.04)	<.001
Age, y		
<65	1 [Reference]	
65-74	−0.01 (−0.03 to 0.00)	.13
≥75	−0.03 (−0.05 to −0.01)	.002
Women	−0.01 (−0.02 to 0.00)	.10
Race and ethnicity		
Black	0.01 (−0.02 to 0.03)	.59
Hispanic	−0.01 (−0.02 to 0.01)	.57
White	1 [Reference]	NA
Other^a^	−0.02 (−0.04 to 0.01)	.21
Income		
<$25 000	1 [Reference]	
$25 000 or more	−0.03 (−0.04 to −0.02)	<.001
Metropolitan area	0.00 (−0.01 to 0.02)	.51
US census regions		
Northeast	1 [Reference]	NA
Midwest	−0.01 (−0.03 to 0.00)	.16
South	−0.03 (−0.05 to −0.02)	<.001
West	0.00 (−0.01 to 0.02)	.75
Dual eligibility for Medicare and Medicaid	0.00 (−0.02 to 0.02)	.91
Use of a language other than English at home	−0.01 (−0.03 to 0.01)	.36
Access to telehealth		
Yes	1 [Reference]	NA
No	0.01 (0.00 to 0.03)	.01
Do not know	0.01 (0.00 to 0.03)	.14
Ability to access basic needs during the pandemic		
Pay rent/mortgage	−0.03 (−0.08 to 0.02)	.28
Get medication	−0.10 (−0.15 to −0.04)	<.001
Get food that is wanted	−0.04 (−0.08 to −0.01)	.02
Get home supplies	−0.08 (−0.10 to −0.05)	<.001
Health conditions		
Hypertension	0.00 (−0.02 to 0.01)	.40
Myocardial infarction	0.00 (−0.02 to 0.02)	.83
Congestive heart failure	0.00 (−0.03 to 0.02)	.69
Stroke	0.01 (−0.01 to 0.02)	.47
High cholesterol	0.00 (−0.01 to 0.01)	.83
Cancer	0.01 (0.00 to 0.02)	.16
Alzheimer disease/dementia	−0.02 (−0.06 to 0.01)	.22
Depression	0.02 (0.00 to 0.03)	.02
Osteoporosis	0.02 (0.01 to 0.03)	.004
Broken hip	−0.03 (−0.05 to 000)	.02
Emphysema/asthma/COPD	0.03 (0.01 to 0.04)	<.001
Diabetes	0.01 (0.00 to 0.02)	.06
Weak immune system	0.05 (0.04 to 0.07)	<.001
Smoking status		
Current	1 [Reference]	
Former	0.02 (0.01 to 0.04)	.01
Never	0.02 (0.01 to 0.04)	.01
**Interview data**		
Week of		
June 7, 2020	1 [Reference]	NA
June 14, 2020	−0.02 (−0.05 to 0.01)	.27
June 21, 2020	−0.03 (−0.06 to 0.01)	.11
June 28, 2020	0.00 (−0.04 to 0.04)	.96
July 5, 2020	−0.04 (−0.09 to 0.00)	.07
July 12, 2020	−0.07 (−0.12 to −0.01)	.01
October 4, 2020	−0.13 (−0.16 to −0.10)	<.001
October 11, 2020	−0.13 (−0.16 to −0.10)	<.001
October 18, 2020	−0.15 (−0.18 to −0.12)	<.001
October 25, 2020	−0.15 (−0.18 to −0.12)	<.001
November 1, 2020	−0.15 (−0.18 to −0.12)	<.001
November 8, 2020	−0.12 (−0.16 to −0.08)	<.001
February 28, 2021	−0.14 (−0.16 to −0.11)	<.001
March 7, 2021	−0.15 (−0.17 to −0.12)	<.001
March 14, 2021	−0.17 (−0.19 to −0.14)	<.001
March 21, 2021	−0.15 (−0.19 to −0.12)	<.001
March 28, 2021	−0.17 (−0.20 to −0.13)	<.001
April 4-25, 2021	−0.17 (−0.20 to −0.14)	<.001

Abbreviations: COPD, chronic obstructive pulmonary disease; NA, not applicable.

^a^ Includes American Indian or Alaska Native, Asian, Native Hawaiian or other Pacific Islander, or multiracial individuals.

**Figure 3.**
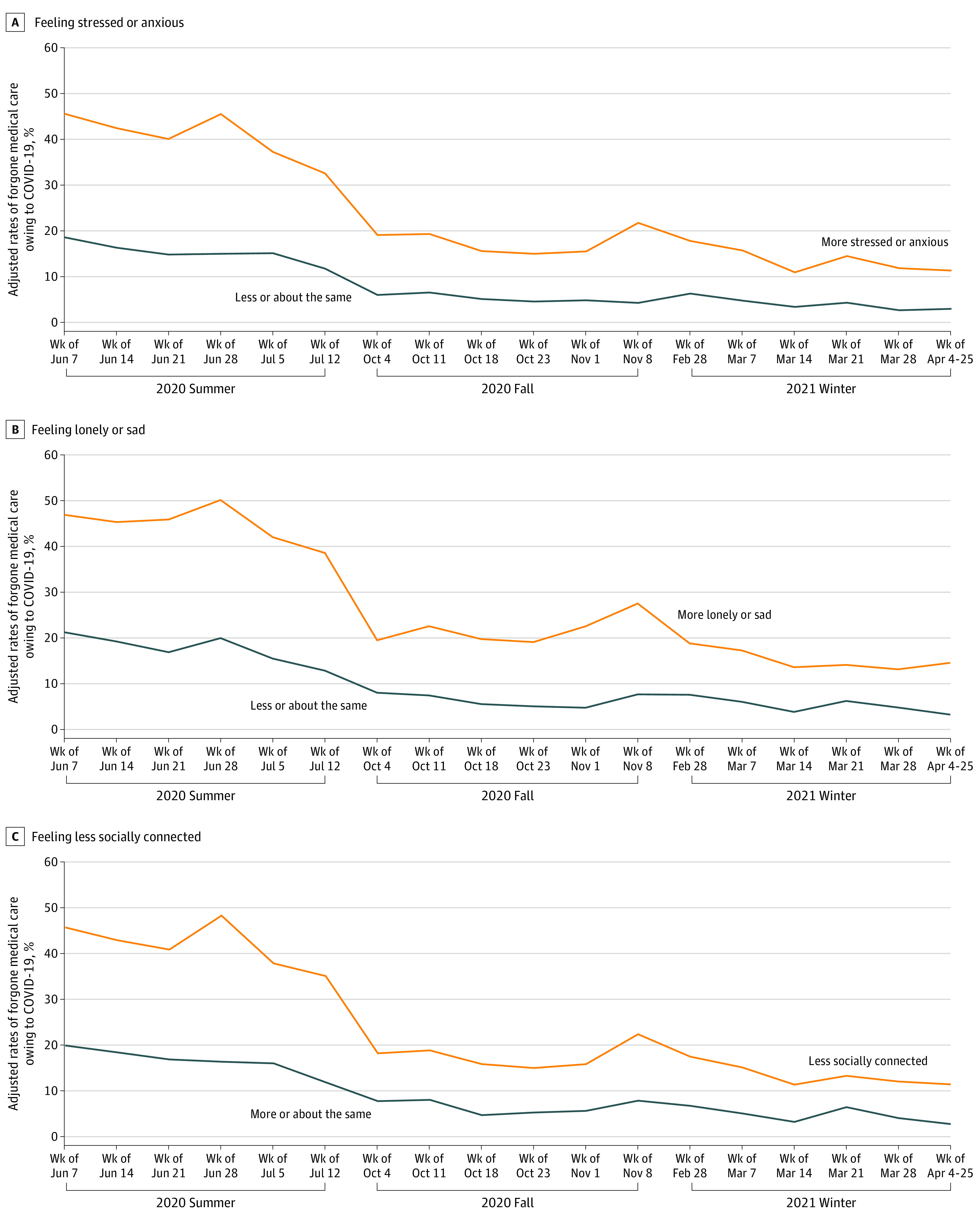
Adjusted Rates of Reported Forgone Medical Care Because of COVID-19 Among Medicare Beneficiaries by Date of Interview and Mental Health Status Adjusted rates were estimated using a linear probability model on forgone medical care while controlling for age, sex, race and ethnicity, income, rural residence, census region of residence, Medicaid eligibility, use of a language at home other than English, access to telemedicine, ability to access basic needs during the pandemic, self-reported health conditions, the date of interview, mental health status, and interaction term between the date of interview and mental health status. Then, we estimated the adjusted rates of the outcome by date of interview and mental health status. For all analyses, we accounted for complex sample design with sampling weights provided by the Medicare Current Beneficiary Survey (MCBS) to generate nationally representative estimates.

The adjusted analysis found that forgone medical care was more prevalent among those who reported mental health problems (Figure 3). The adjusted rates of forgone medical care because of COVID-19 were substantially higher among those who reported feeling more stressed or anxious than those who did not (27.3% [95% CI, 23.4%-31.1%] vs 18.6% [95% CI, 15.8%-21.4%] during the week of July 7, 2020), among those who reported feeling more lonely or sad than those who did not (26.0% [95% CI, 21.3%-30.8%] vs 21.3% [95% CI, 18.7%-23.9%] during the week of July 7, 2020), and among those who reported feeling less socially connected than those who did not (26.1% [95% CI, 22.1%-30.1%] vs 20.0% [95% CI, 17.3%-22.8%] during the week of July 7, 2020). In all groups, the adjusted rates of forgone medical care because of COVID-19 decreased from the week of June 7, 2020, to the week of April 4 to 25, 2021, but the magnitude of the decrease was larger among those who reported mental health problems. However, the gaps in the likelihood of forgone medical care persisted during the weeks of April 4 to 25, 2021 (8.4% [95% CI, 4.6%-12.3%] vs 3.0% [95% CI, 1.1%-5.0%] for those who reported feeling more stressed or anxious vs those who did not, 11.5% [95% CI, 5.5%-17.6%] vs 3.4% [95% CI, 1.6%-5.2%] for those who reported feeling more lonely or sad vs those who did not, and 8.8% [95% CI, 4.8%-12.7%] vs 2.9% [95% CI, 1.0%-4.8%] for those who reported feeling less socially connected vs those who did not). We found little evidence that mental health status changed during the study period (eFigures 3 and 4 in the Supplement).

## Discussion

Using nationally representative survey data for Medicare beneficiaries, we examined trends in and reasons for forgone medical care during the COVID-19 pandemic. First, we identified about one-fourth of Medicare beneficiaries who reported forgoing medical care during Summer 2020. We also found that the trend in forgone care decreased during later stages of the COVID-19 pandemic. A substantial decrease was found in Summer 2020 before a vaccine for COVID-19 was widely available in the US. This study adds to the literature by identifying factors that contributed to forgone care in a nationally representative population of Medicare beneficiaries who had stable health insurance during the pandemic. Physician factors, such as closed physician’s offices and availability of appointments, were identified as major contributors to forgone care during the pandemic in this population. Patient factors, such as fear of COVID-19 exposure, were also contributing factors, which is consistent with some prior studies.^7,8,9,10,11^

Our estimate for forgone medical care during the pandemic was lower than the estimate from prior work (40.9%).^7^ There may be multiple explanations for this discrepancy in findings, for example, differences in the timing of measurement (eg, right after the onset of the pandemic or later), study population (eg, adults or older adults), and questions asked (eg, forgone medical care overall or forgone medical care because of COVID-19). Prior research showed that the number of weekly outpatient visits was the lowest in April 2020, but gradually increased since then and became close to the typical-year trend in September 2020.^26^ However, as our data do not include the period of the initial onset pandemic, we could not estimate the degree to which forgone care because of COVID-19 decreased among Medicare beneficiaries.

This study’s results also suggested that forgone medical care was more common among those who reported mental health problems. This aligns with findings from prior research that mental health was associated with unmet or forgone medical care before the COVID-19 pandemic.^21^ The study results found that the likelihood of forgone medical care was especially higher among those who reported mental health problems than those who did not during the early study period, possibly suggesting that the pandemic disproportionately increased forgone medical care among Medicare beneficiaries with mental health problems. The association between forgone medical care and mental health problems is likely to be bidirectional, although evidence is limited.^25^ Identifying this association is a high priority given that the policy implications are different in each direction.

Although forgone medical care decreased over time in those who reported mental health problems and those who did not, the gaps in the likelihood of forgone medical care persisted. This finding may be partly attributable to gaps in Medicare mental health coverage. For example, Medicare beneficiaries are limited to 190 days of inpatient psychiatric hospice care. However, those with chronic mental illness may exceed this limit, resulting in high out-of-pocket costs for needed inpatient care. Also, payments for mental health clinicians are restricted; thus, receipt of mental care is more likely to rely on the availability of mental health clinicians. These findings may suggest the need to develop policies to provide additional resources and/or support to address mental health problems, especially in the context of the COVID-19 pandemic.

### Limitations

This study has several limitations. First, we relied on survey data, and thus our findings may be subject to self-reporting errors. Second, our study analyzed trends in forgone medical care, but it was limited to a relatively short period. Third, state and local governments implemented various policies to respond to the COVID-19 pandemic, which may have affected access to care. As the MCBS Public Use File does not contain information on state residency, we could not account for differences in policies at the state or county level. Fourth, we assumed that Medicare beneficiaries experienced forgone medical care on the date of the interview. However, there may be a discrepancy between when Medicare beneficiaries were interviewed and had forgone medical care. Fifth, it is possible that some beneficiaries were interviewed more than once. As we could not identify them through the MCBS Public Use File, we treated the data for each wave as an independent cross-sectional survey. Sixth, our measure of mental health status does not rely on clinical mood or disorders and thus may capture different aspects of mental health problems. Thus, our findings should be interpreted with caution. Finally, we used a cross-sectional study design, but we could not control for all potential factors that might be associated with forgone medical care. Because our findings are associations and not causal, they should be interpreted cautiously.

## Conclusions

The results of this cross-sectional survey study suggest that public health emergencies, such as pandemics, may exacerbate existing barriers to care and cause patients to delay needed care. Factors unique to the pandemic included closed physician’s offices, reduced appointment availability, and patient fear of contagion. Medicare beneficiaries who are experiencing heightened mental health problems associated with the COVID-19 pandemic appear to be particularly vulnerable to forgone medical care. Policy makers must continue to identify effective means of meeting the forgone care backlog and maintaining continuity of care, especially for those with mental health problems.

## Notes

Supplement. eAppendix. Questions from the survey instrument eTable. Sample characteristics eFigure 1. Unadjusted rates of forgone medical care due to COVID-19 among Medicare beneficiaries by date of interview eFigure 2. Adjusted rates of forgone medical care due to COVID-19 among nondual Medicare beneficiaries by date of interview eFigure 3. Unadjusted rates of mental health status among Medicare beneficiaries by date of interview eFigure 4. Adjusted rates of mental health status among Medicare beneficiaries by date of interview
