# Supplementary material for: Trends in Self-reported Forgone Medical Care Among Medicare Beneficiaries During the COVID-19 Pandemic
Source: JAMA Health Forum. 2021 Dec 30;2(12):e214299. doi: 10.1001/jamahealthforum.2021.4299 (PMC8796880; doi:10.1001/jamahealthforum.2021.4299)
Supplement: Supplement. — eAppendix. Questions from the survey instrument eTable. Sample characteristics eFigure 1. Unadjusted rates of forgone medical care due to COVID-19 among Medicare beneficiaries by date of interview eFigure 2. Adjusted rates of forgone medical care due to COVID-19 among nondual Medicare beneficiaries by date of interview eFigure 3. Unadjusted rates of mental health status among Medicare beneficiaries by date of interview eFigure 4. Adjusted rates of mental health status among Medicare beneficiaries by date of interview [file jamahealthforum-e214299-s001.pdf]

Supplemental Online Content

Park S, Stimpson JP. Trends in self-reported forgone medical care among Medicare beneficiaries during the COVID-19 pandemic. *JAMA Health Forum*. 2021;2(12):e214299. doi:10.1001/jamahealthforum.2021.4299

eAppendix. Questions from the survey instrument
eTable. Sample characteristics
eFigure 1. Unadjusted rates of forgone medical care due to COVID-19 among Medicare beneficiaries by date of interview
eFigure 2. Adjusted rates of forgone medical care due to COVID-19 among nondual Medicare beneficiaries by date of interview
eFigure 3. Unadjusted rates of mental health status among Medicare beneficiaries by date of interview
eFigure 4. Adjusted rates of mental health status among Medicare beneficiaries by date of interview

This supplemental material has been provided by the authors to give readers additional information about their work.

© 2021 Park S et al. *JAMA Health Forum.*

eAppendix.

Questions from the survey instrument

Our outcome variable was (self-reported) forgone medical care due to COVID-19. Participants were asked the following question: “since the coronavirus pandemic began, have you needed medical care for something other than coronavirus, but not gotten it because of the pandemic?” We created a binary indicator for forgone medical care due to COVID-19. We included three measures of mental health status during the COVID-19 pandemic relative to the pre-pandemic (stress/anxiety, loneliness/sadness, and social connection). Participants were asked the following question: “since the coronavirus outbreak began, have you felt ...?” We categorized responses as more or not (including “about the same” or “less”) for stress/anxiety and loneliness/sadness and as less or not (including “about the same” or “more”) for social connection.

© 2021 Park S et al. *JAMA Health Forum.*

eTable. Sample characteristics

Variables	N (%) (n=23115 and weighted n = 129076155)
Age	
<65 years	3565 (15.4)
65-74 years	9083 (39.3)
75 + years	10467 (45.3)
Female	13036 (56.4)
Race/ethnicity	
Black	2024 (8.8)
Hispanic	2144 (9.3)
White	17855 (77.2)
Other[a]	1092 (4.7)
Income	
Less than $25000	7608 (32.9)
$25000 or more	15507 (67.1)
Metro	17778 (76.9)
US census regions	
Northeast	4124 (17.8)
Midwest	5393 (23.3)
South	8816 (38.1)
West	4782 (20.7)
Dual eligibility for Medicare and Medicaid	3833 (16.6)
Use of other language other than English spoken at home	2422 (10.5)
Access to telehealth	
Yes	14093 (61.0)
No	3573 (15.5)
Do not know	5449 (23.6)
Ability to access basic needs during the pandemic	
Able to pay rent/mortgage	22772 (98.5)
Able to get medication	22785 (98.6)
Able to get food wanted	22373 (96.8)
Able to get home supplies	21574 (93.3)
Health conditions	
Hypertension	15138 (65.5)
Myocardial infarction	2305 (10.0)
Congestive heart failure	1447 (6.3)
Stroke	2117 (9.2)
High cholesterol	15324 (66.3)
Cancer	4818 (20.8)
Alzheimer's disease/dementia	364 (1.6)
Depression	6068 (26.3)
Osteoporosis	4556 (19.7)
Broken hip	792 (3.4)
Emphysema/asthma/COPD	4534 (19.6)
Diabetes	7557 (32.7)
Weak immune system	4029 (17.4)
Smoking status	
Current smoker	2512 (10.9)

© 2021 Park S et al. *JAMA Health Forum.*

Former smoker	10820 (46.8)
Never smoked	9783 (42.3)
Interview date	
Week of June 7, 2020	1859 (8.0)
Week of June 14, 2020	2684 (11.6)
Week of June 21, 2020	1539 (6.7)
Week of June 28, 2020	973 (4.2)
Week of July 5, 2020	682 (3.0)
Week of July 12, 2020	297 (1.3)
Week of October 4, 2020	1293 (5.6)
Week of October 11, 2020	1746 (7.6)
Week of October 18, 2020	1285 (5.6)
Week of October 25, 2020	1154 (5.0)
Week of November 1, 2020	924 (4.0)
Week of November 8, 2020	486 (2.1)
Week of February 28, 2021	2493 (10.8)
Week of March 7, 2021	2539 (11.0)
Week of March 14, 2021	1378 (6.0)
Week of March 21, 2021	711 (3.1)
Week of March 28, 2021	453 (2.0)
Weeks of April 4-25, 2021	619 (2.7)
Mental health status	
More stressed or anxious	9178 (39.7)
More lonely or sad	4964 (21.5)
Less socially connected	8605 (37.2)
Forgone medical care	
Any care	2661 (11.5)
Urgent care	140 (0.6)
Surgery	430 (1.9)
Diagnostics	769 (3.3)
Prevention	932 (4.0)
Check up	912 (3.9)
Dental	992 (4.3)
Vision	605 (2.6)
Hearing	144 (0.6)

[a] Include American Indian or Alaska Native, Asian, Native Hawaiian or other Pacific Islander, or those with two or more races.

© 2021 Park S et al. *JAMA Health Forum.*

eFigure 1. Unadjusted rates of forgone medical care due to COVID-19 among Medicare beneficiaries by date of interview.

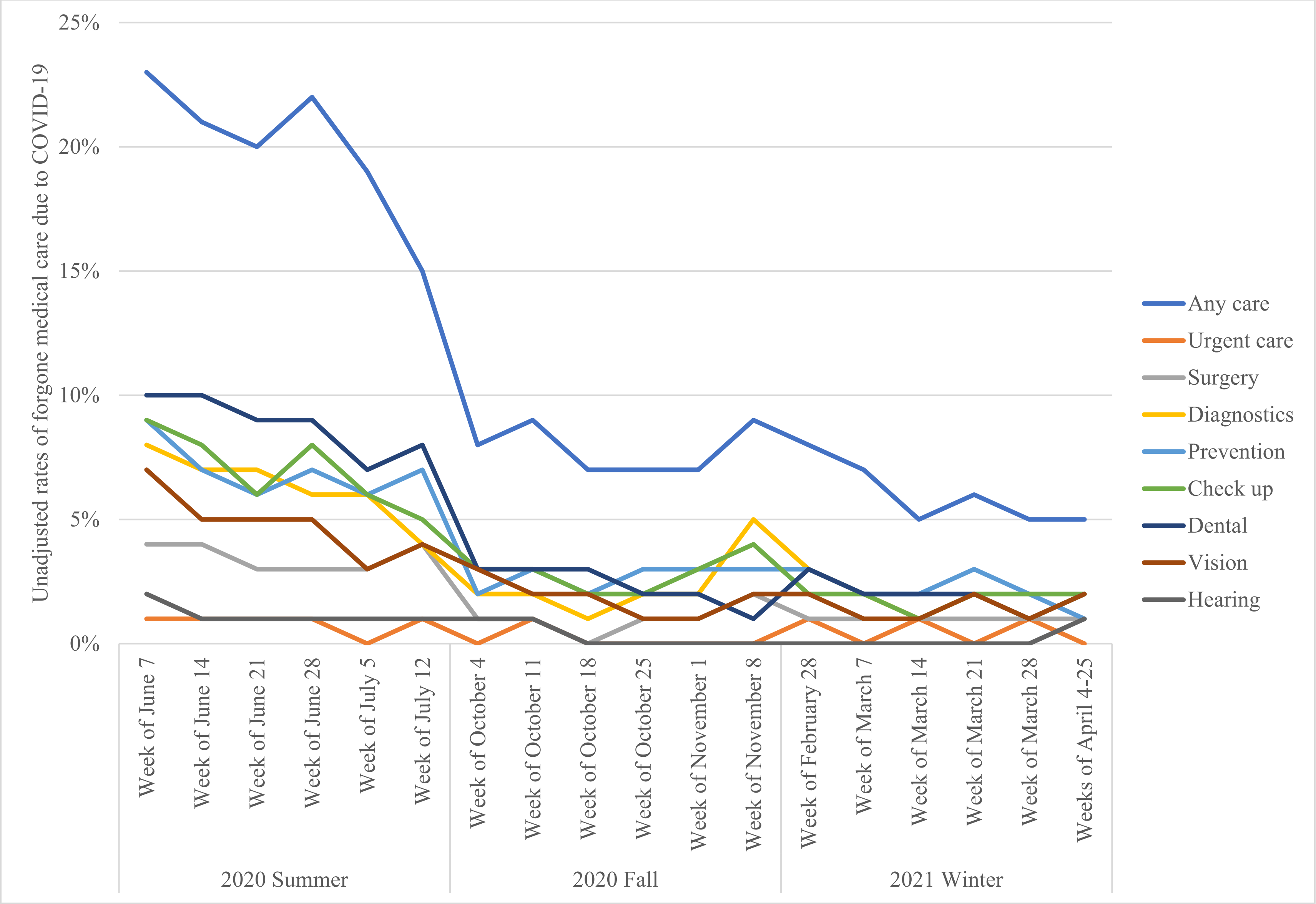

© 2021 Park S et al. *JAMA Health Forum.*

eFigure 2. Adjusted rates of forgone medical care due to COVID-19 among nondual Medicare beneficiaries by date of interview.

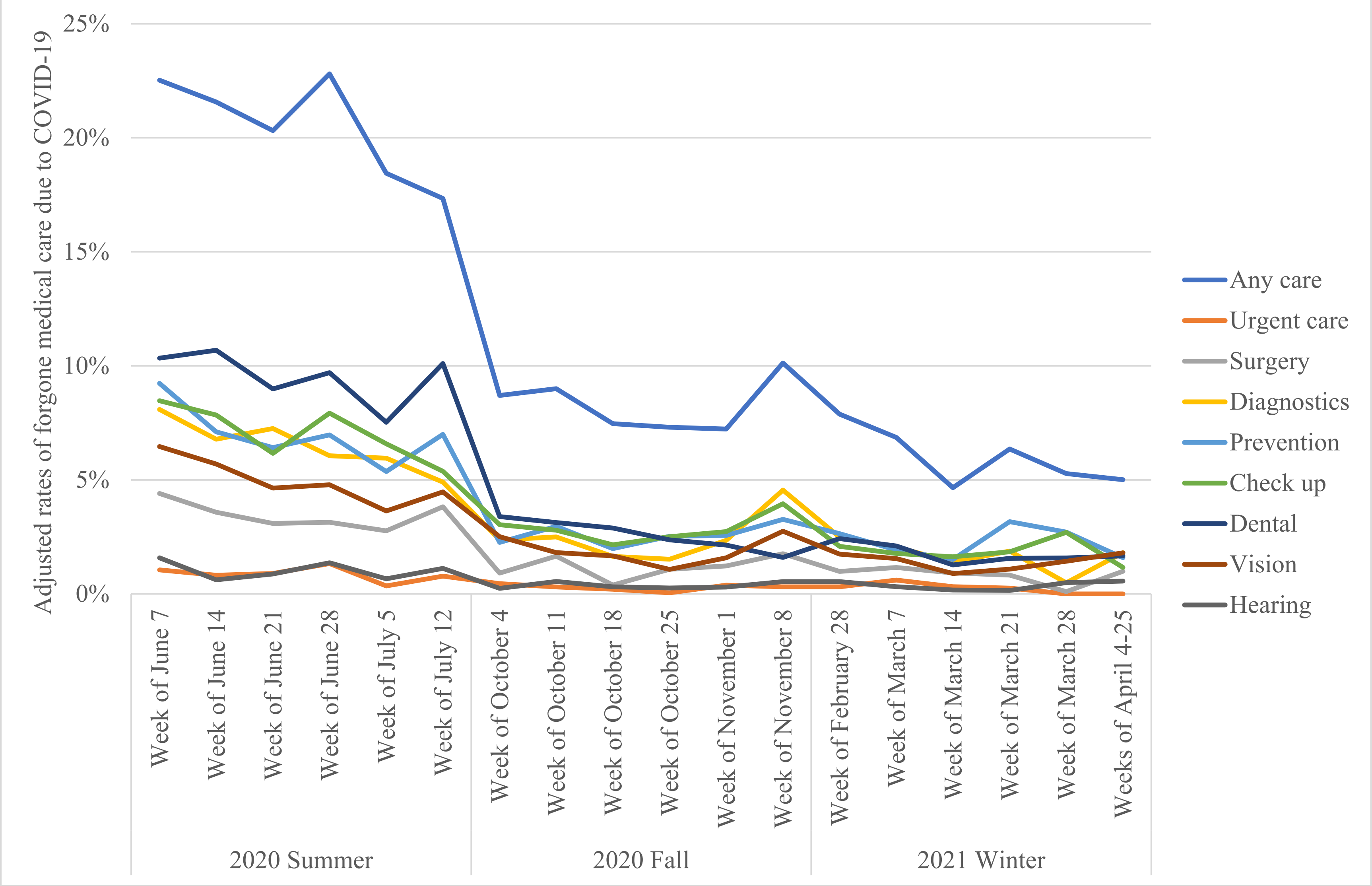

© 2021 Park S et al. *JAMA Health Forum.*

eFigure 3. Unadjusted rates of mental health status among Medicare beneficiaries by date of interview.

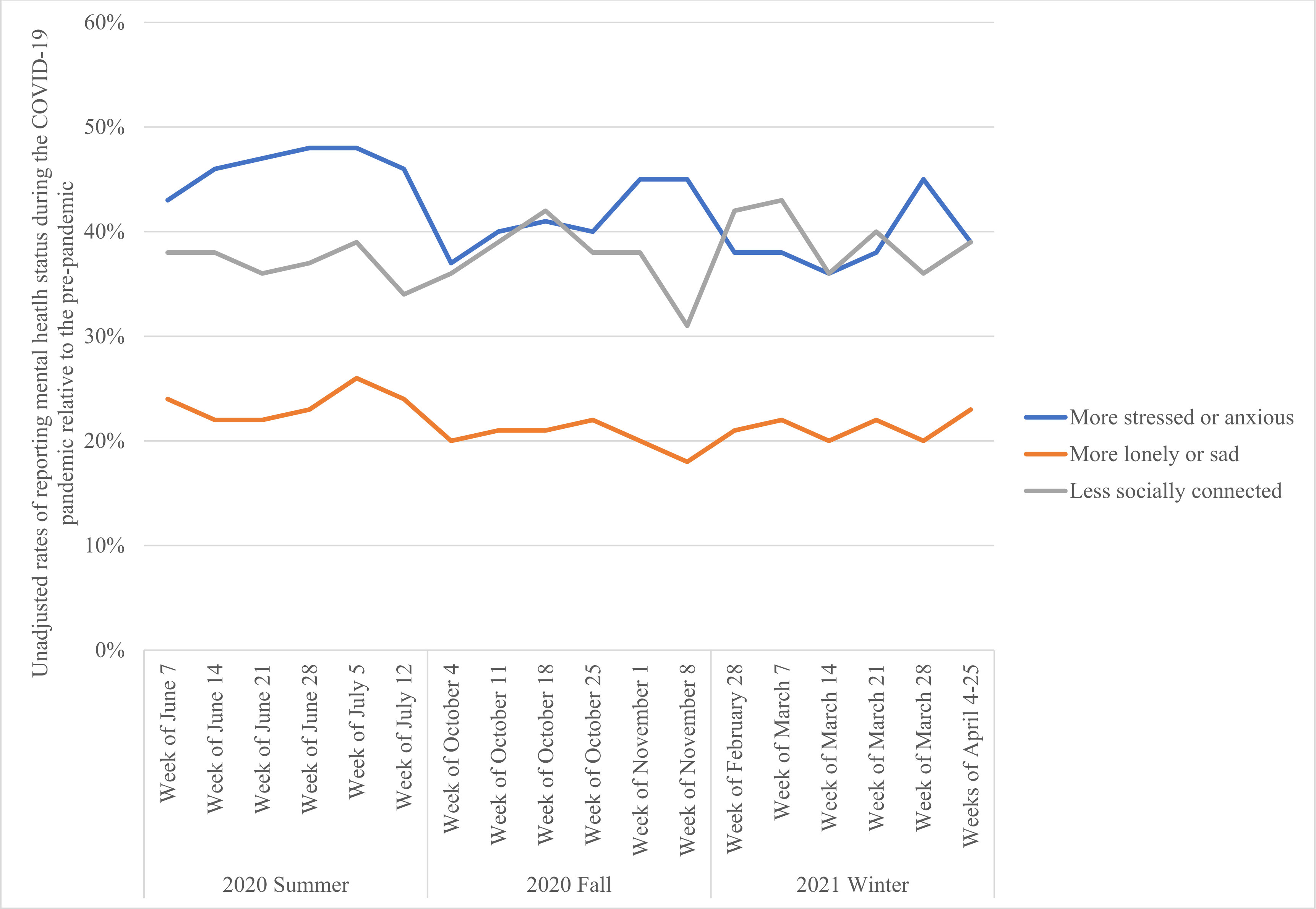

© 2021 Park S et al. *JAMA Health Forum.*

eFigure 4. Adjusted rates of mental health status among Medicare beneficiaries by date of interview.

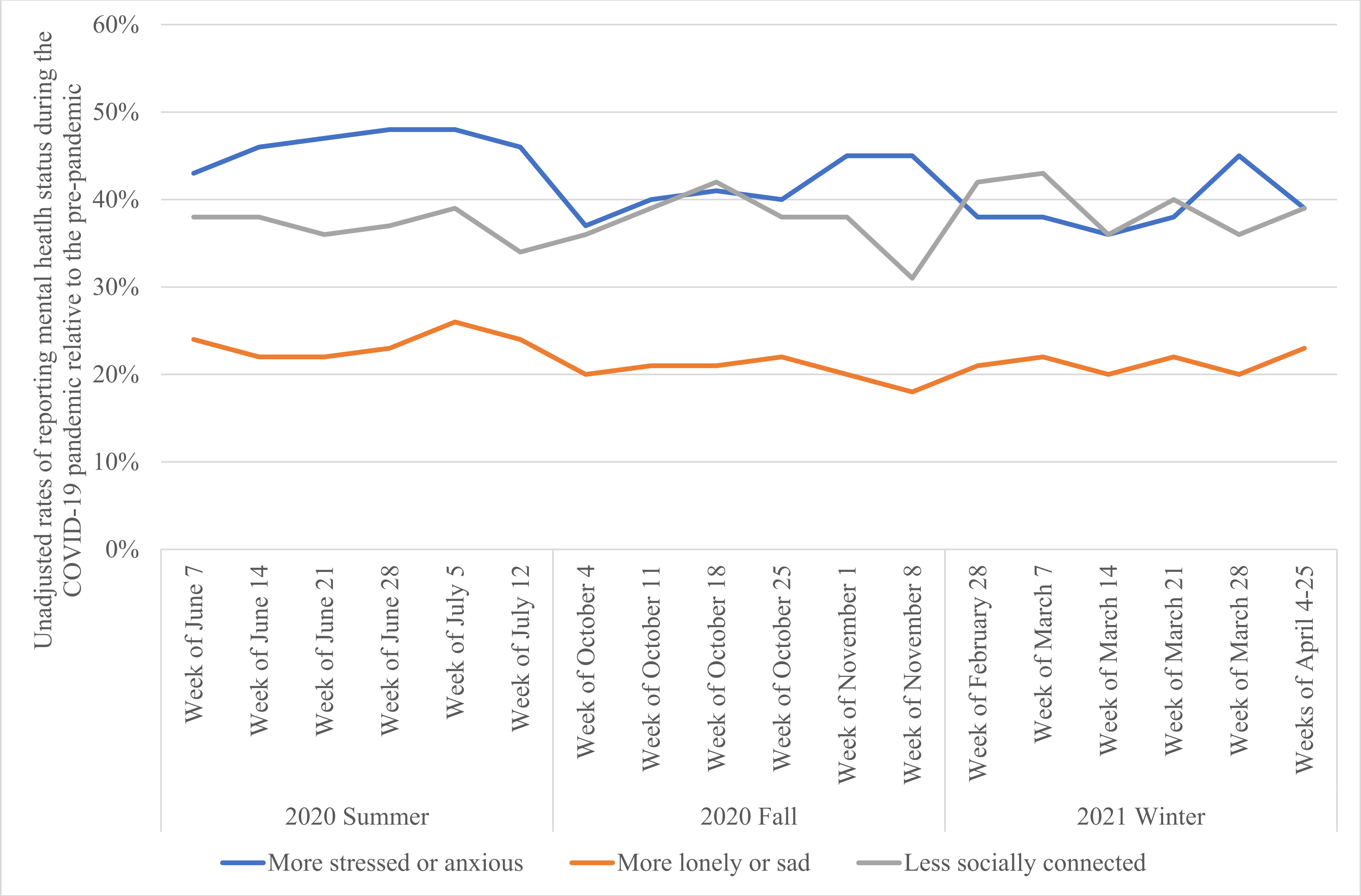

© 2021 Park S et al. *JAMA Health Forum.*
